# Elderly onset of MELAS in a male: A case report

**DOI:** 10.3389/fneur.2022.1018529

**Published:** 2022-12-01

**Authors:** Sheng-Peng Diao, Song-Fa Chen, Ai-Qun Liu, Zhi-Hua Zhou, Zhong-Xing Peng, Ming-Fan Hong

**Affiliations:** Department of Neurology, College of Clinical Medicine, The First Affiliated Hospital, Guangdong Pharmaceutical University, Guangzhou, China

**Keywords:** MELAS, stroke-like episodes, magnetic resonance angiography (MRA), migraine, magnetic resonance imaging (MRI)

## Abstract

**Background:**

Mitochondrial encephalomyopathy with lactic acidosis and stroke-like episodes (MELAS) is one of the most common maternally inherited mitochondrial diseases which rarely affects elderly people.

**Case presentation:**

We reported the case of a 61-year-old male patient with MELAS. He was experiencing acute migraine-like headaches as the first symptoms. Laboratory data showed elevated lactate and creatine kinase levels. Brain magnetic resonance imaging (MRI) found a high signal intensity lesion in the left occipital-temporal-parietal lobe on diffusion-weighted imaging (DWI). Magnetic resonance angiography (MRA) revealed reversible vasoconstriction of the middle cerebral arteries and superficial temporal arteries. A muscle biopsy suggested minor muscle damage. A genetic study revealed a mitochondrial DNA A3243G mutation.

**Conclusion:**

Elderly onset of MELAS is rare and easily misdiagnosed as an ischemic stroke. MELAS with the onset of stroke-like episodes should be considered in adult or elderly patients with imaging findings that are atypical for cerebral infarction. The use of multimodal MRI in the clinical diagnosis of MELAS could be extremely beneficial.

## Background

Mitochondrial encephalomyopathy with lactic acidosis and stroke-like episodes (MELAS) is one of the most common maternally inherited mitochondrial diseases (1). MELAS is typically characterized by stroke-like episodes and hyperlactic acidemia. Nevertheless, only approximately half of patients show typical clinical manifestations, with significant heterogeneity in genetics and clinical manifestations (2, 3), which leads to difficulty in diagnosis and even misdiagnosis.

Only 1–6% of patients develop the disease after 40 years of age (4). It is even rare in patients after 60 years of age, and there have been only a few cases reported in female patients (5–7). In this report, we described the case of a 61-year-old male patient with MELAS. The clinical and imaging features of the patient helped us in understanding the clinical manifestations of MELAS in the elderly and making a precise diagnosis.

## Case presentation

### First attack

A previously healthy 61-year-old male patient presented with sudden onset of a left-sided migraine-like headache (December 26, 2019) and developed a right-sided migraine-like headache after 2 days. He was immediately admitted to a local hospital for treatment. The results of the brain CT scan (data not shown) showed acute cerebral infarction of the left occipital-parietal lobe, and he was diagnosed with acute ischemic cerebral infarction. The patient was then treated with aspirin (100 mg/day), atorvastatin (20 mg/day), and citicoline (0.6 g/day). However, he had weakness in his right limb and could not walk for 7 days after onset. He subsequently showed emotional irritability and hallucinations 8 days after onset. There was no history of blurred vision, hearing impairment, migraine headaches, or gastroenteritis. In addition, there was no history of diabetes, hypertension, or a family history of stroke, and no history of smoking or drinking. No one in his family had a similar medical history.

The patient was admitted to our hospital 11 days after onset (6 January 2020). On physical examination, his blood pressure was 112/69 mmHg, and he had lethargy and a lag in response with slow speech. He also had right homonymous hemianopia, which was shown by a slight wrinkle on the right frontal, a slight nasolabial groove on the right, and his mouth was skewed to the left with a tongue extended into the middle. The patient was given grade 1 for right upper extremity strength, grade 3 for right lower extremity strength, and grade 5 for left extremity strength, with reduced tendon reflex of the limbs. The pathological sign was negative. His score was 10 according to the United States National Institutes of Health Stroke Scale (NIHSS).

After 12 days of onset, clinical chemistry analysis revealed fasting blood glucose levels of 6.6 mmol/L, glycated hemoglobin levels of 6.1%, creatine kinase levels of 448 U/L, high sensitivity C-reactive protein levels of 42.1 mg/L, and arterial blood lactic acid levels of 3.3 mmol/L. The results of routine hematological tests, homocysteine, blood lipids, four coagulation tests, erythrocyte sedimentation rate, antinuclear antibodies, anti-cardiolipin antibodies, anti-neutrophil antibodies, protein C, and protein S were all within the normal range. The results of the cerebrospinal fluid (CSF) assay showed a value of 4.13 mmol/L for lactic acid, and routine biochemistry did not reveal any abnormalities.

**Figure 1:**
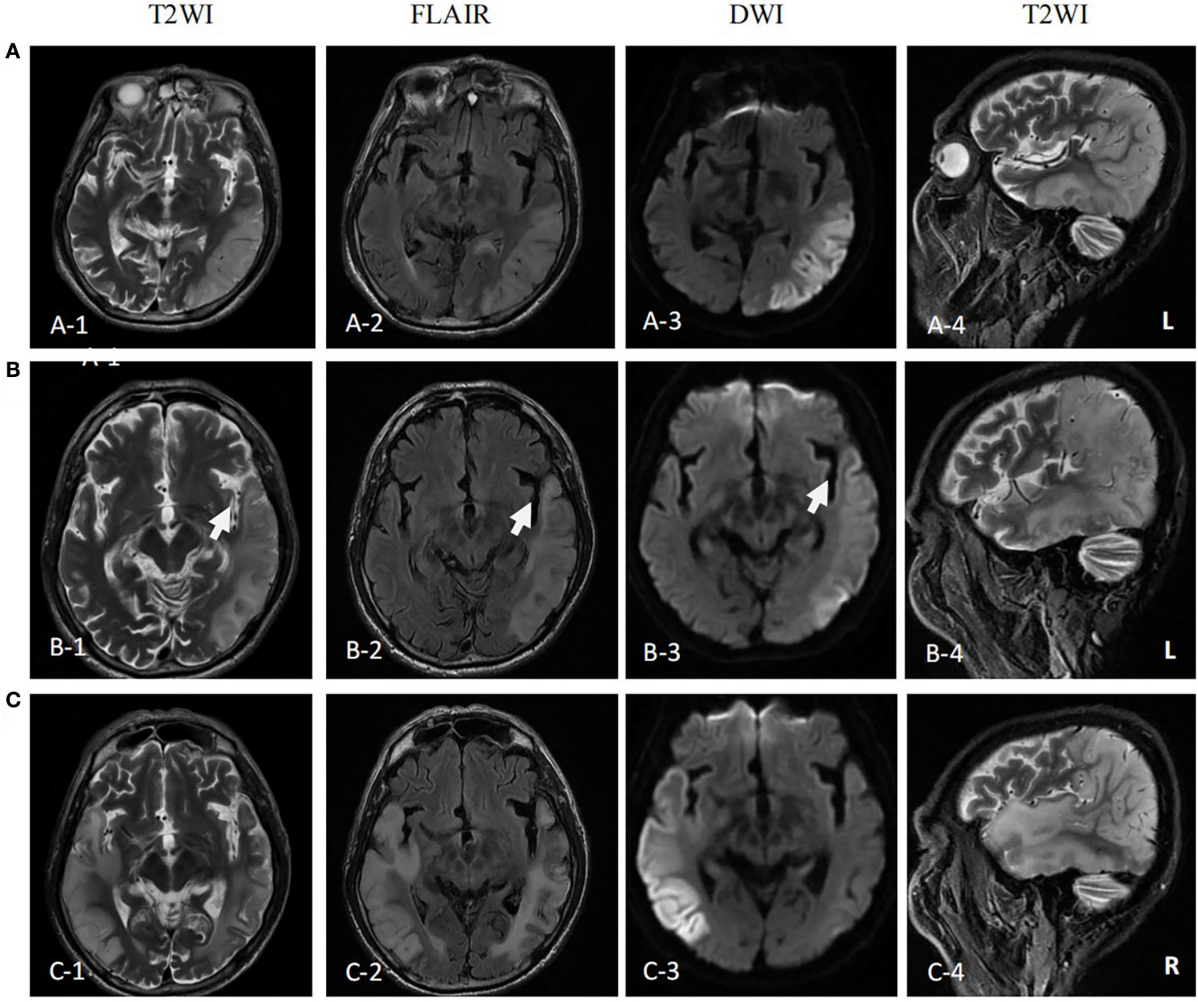
MR images of the patient. **(A)** MR images performed on the first attack (12 days after onset) showed high signal intensity in occipital-temporal-parietal lobe hyperextension on T2-weighted, fluid attenuation inversion recovery (FLAIR), and diffusion-weighted imaging (DWI). **(B)** MR images performed on the remission (27 days after the first onset) showed a high signal intensity of DWI, FLAIR, and T2WI of the left occipital-temporal-parietal lobe was reduced. The focus of the temporal lobe is enlarged (arrow). **(C)** MR images performed on the recurrence (68 days from the first onset) showed new high signal intensity in the right occipital-temporal-parietal lobe hyperextension on T2-weighted, FLAIR, and DWI.

**Figure 2:**
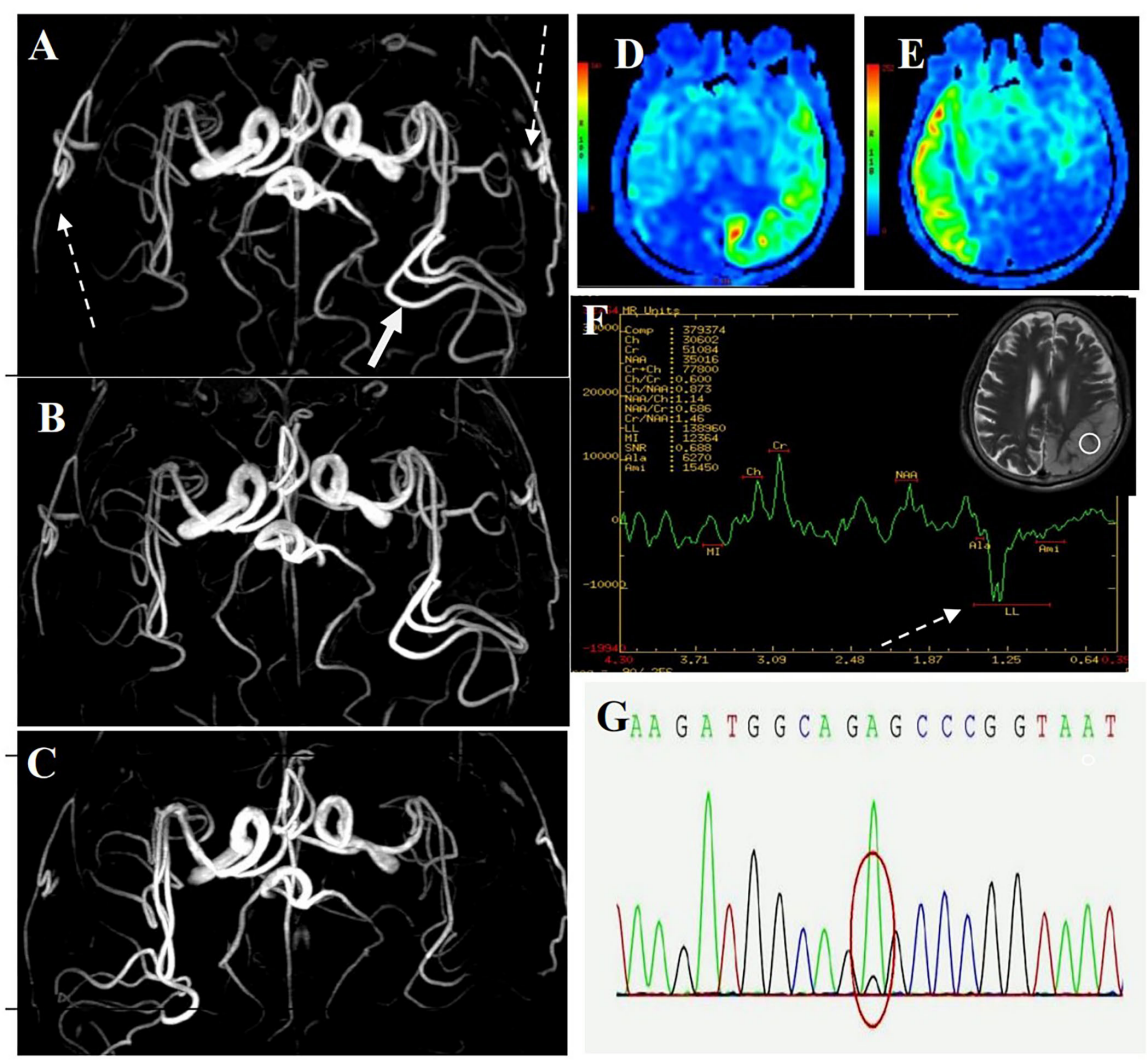
MR images of the patient. **(A)** MRA image of first onset period, 12 days after onset. MRA showed dilation of the left middle cerebral artery (long-tail arrow), with apparent dilation of superficial temporal arteries (long dotted arrow). **(B)** MRA image of remission period, 27 days after onset. MRA showed that the superficial temporal arteries were retracted. **(C)** MRA images of recurrence period, 68 days after onset. MRA showed that the left middle cerebral artery appeared normal, and the right middle cerebral artery was dilated. **(D)** ASL image of first onset period, 12 days after onset. ASL showed hyperperfusion in the left occipital-temporal-parietal focal areas. **(E)** ASL images of recurrence period, 68 days after onset. ASL showed hyperperfusion in the right occipital-temporal-parietal focal regions. **(F)** MRS image of first onset period, 12 days after onset. MRS showed a double inverted lactate peak at 1.33 ppm (short dotted arrow). **(G)** Genotypic detection analysis showed mitochondrial mutation (m.3243A >G).

**Figure 3:**
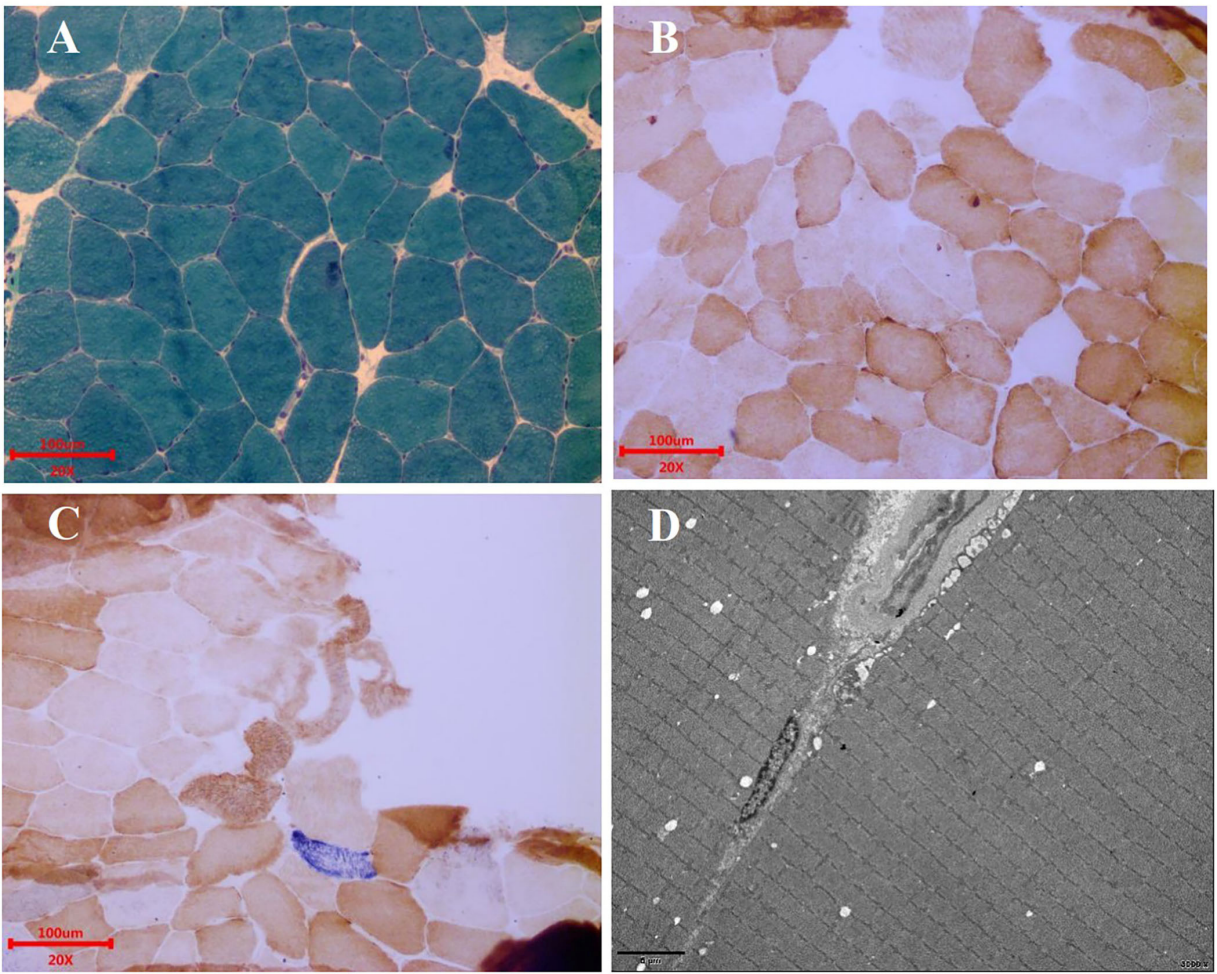
Muscle biopsy. **(A)** MGT staining showed no ragged red fibers. Scale bars: 100 μm. **(B)** COX staining showed no significant decrease in myofibrillase activity. Scale bars: 100 μm. **(C)** SDH/COX double staining found one blue fiber. Scale bars: 100 μm. **(D)** Under the electron microscope, the basic arrangement of myofibrils was regular, the sarcolemma was shrunken, and no special ultrastructural and pathological changes were found. Scale bars: 5 μm.

The first magnetic resonance imaging (MRI) examination was performed 12 days after onset (7 January 2020) (Figure 1A), and diffusion-weighted imaging (DWI) showed a high signal intensity (Figures 1A, **3**). Magnetic resonance angiography (MRA) showed dilation of the left middle cerebral artery, posterior cerebral artery, and bilateral superficial temporal arteries (Figure 2A). Arterial spin labeling (ASL) indicated hyperperfusion in the left occipital-temporal-parietal focal areas (Figure 2D). There was a double inverted lactate peak at 1.33 ppm detected by magnetic resonance spectroscopy (MRS) (Figure 2F).

Electroencephalogram (EEG) monitoring revealed several high amplitudes and sharp slow-wave activities in the left and right hemispheres, varying during waking and sleeping. A muscle biopsy was performed on the patient. The modified gomori trichrome (MGT) staining was negative (Figure 3). The succinate dehydrogenase (SDH)/c oxidase (COX) double staining showed a blue fiber, no positive result of blood vessel wall strength, and no obvious abnormality of muscle fiber under the electron microscope (Figure 3) (8). Next, 3 ml of peripheral venous blood from the patient was drawn for genotypic detection analysis, and the results indicated mitochondrial 3243A>G mutation (Figure 2G), which is a pathogenic mutation (2). The proportion of the m.3243A>G mutation in blood was 15%.

### Remission

The patient was diagnosed with MELAS by gene testing. After initial treatment (adenosine triphosphate disodium 60 mg/day, coenzyme Q10 30 mg/day, and L-arginine 20 g/day), the patient's condition showed significant improvement, and he was able to live independently. The second MRI examination was performed 27 days after the first onset (22 January 2020). DWI showed that the high signal intensity of the left occipital-temporal-parietal lobe was reduced (Figure 1B). In addition, MRA showed that the bilateral superficial temporal arteries were retracted (Figure 2B).

### Recurrence

Unfortunately, the patient relapsed 68 days after the first onset (3 March 2020). There was a twitch on the left limb of the patient; his limbs were weak, and he was unable to walk. The third MRI examination was performed on 3 March 2020. DWI revealed a high signal intensity (Figure 1C), and ASL showed hyperperfusion in the right occipital-temporal-parietal focus areas (Figure 2E). In addition, according to MRA, the left middle cerebral artery was normal, whereas the right middle cerebral artery was dilated (Figure 2C). His limb convulsions did not recur after treatment with lamotrigine (50 mg/day), coenzyme Q10 (30 mg/day), and L-arginine (20 g/day), and his limb muscle strength weakness was improved, but he developed dementia and currently requires family care.

## Discussion

Mitochondrial encephalomyopathy with lactic acidosis and stroke-like episodes is the most common clinical form of mitochondrial encephalomyopathy, with stroke-like episodes and hyperlactic acidemia as the primary clinical features, which was first reported by Pavlakis et al. (9). The clinical manifestations of MELAS are usually sudden stroke-like symptoms such as limb weakness, cortical blindness, mental disturbance, mental retardation, epilepsy, headache, and neural deafness (10). MELAS is common in adolescents and rarely seen in the elderly. Some scholars believe that MELAS with onset in over 50-year-old patients is seldom seen and has atypical clinical manifestations (11), making it difficult to diagnose. Only a few cases of this disease have been reported in older women, and there is less knowledge about this disease in the elderly (5–7). There are no case reports of MELAS in elderly men, and one of the factors that make diagnosing MELAS in geriatric-onset patients difficult is the lack of adequate knowledge about MELAS in men aged above 60 years. We herein presented the case of a 61-year-old male patient with MELAS.

As the first symptom, the older man experienced acute migraine-like headaches. The CT scan of the left occipital-parietal lobe at the local hospital revealed low-density lesions. At our hospital, a hyperintense lesion was seen in the left occipital-temporal-parietal lobe on a brain MRI.

This patient was easily misdiagnosed with ischemic cerebrovascular disease. However, MRA did not indicate stenosis or occlusion of the corresponding cerebral responsible vessels. As a result, we suspected the diagnosis of ischemic cerebral infarction and other diseases, such as MELAS. Then, laboratory data showed elevated lactate and creatine kinase levels, and genetic analysis revealed a mitochondrial DNA A3243G mutation. MELAS was confirmed after the patient met the clinical diagnostic criteria (12).

This case has some essential and specific MRA imaging features. MRA revealed reversible vasoconstriction of the middle cerebral arteries and superficial temporal arteries bilaterally. In patients presenting with the acute stage of MELAS, the accumulation of lactate in the lesion results in local arterial dilatation (12). Some blood vessels suffer from chronic damage to the vessel wall, episodic spasm, and intimal hyperplasia as the disease progresses. The angiogram showed normalization progression and reduction in blood vasculature (13). The cerebral blood vessels in the focal area dilated during the initial attack and recurrence stages and retracted during remission. However, there was no such vascular change during acute ischemic stroke. MELAS can be diagnosed and evaluated using MRA, which can detect the cerebrovascular imaging characteristics of MELAS.

Acute migraine-like headaches were the first symptoms that the patient experienced. MRA indicated apparent dilation of the bilateral superficial temporal arteries in the acute phase. The headache gradually disappeared after the treatment. Subsequent MRA showed that the bilateral superficial temporal arteries were slowly retracted and appeared normal. Migraine attacks are thought to be caused by an increase in blood flow through the superficial temporal artery during the acute phase of migraine (14, 15). In recent years, mitochondrial dysfunction has been proven to play an essential role in the pathogenesis of migraine (16). The patient was found to have a mitochondrial DNA A3243G point mutation. The findings of this case suggest that mitochondrial dysfunction may contribute to migraine pathogenesis.

Myopathic symptoms are also manifestations of MELAS, mainly including myasthenia, myalgia, and exercise intolerance (8). The patient underwent a muscle biopsy, and MGT staining was found negative. SDH/COX double staining showed a blue fiber. There was no positive result of blood vessel wall strength and no obvious abnormality of muscle fiber under the electron microscope. A muscle biopsy suggested minor muscle damage. The possibility of missed diagnoses and delayed treatment exists. However, there is a limited correlation between muscle biopsies and the diagnosis of MELAS (17). The presence of mitochondrial disease cannot be ruled out by a normal muscle biopsy (18). In contrast, gene detection plays a vital role in diagnosing MELAS when muscle biopsy fails to diagnose it, and it is the diagnostic gold standard for MELAS (19).

This patient was detected to carry the m.3243A>G mutation, a pathogenic variant of MELAS. The proportion of the m.3243A>G mutation in the blood test was low (15%). More than 80% of the patients have the mitochondrial DNA (mtDNA) 3243A>G mutation (4). Patients with MELAS have obvious heterogeneity and various clinical manifestations, and even monozygotic twins with the m.3243A>G mutation can also be found. The age of onset and clinical phenotype are different (20). Studies have shown that the mtDNA mutation rate of patients with MELAS having ragged red fibers is significantly higher than that of patients without ragged red fibers (18). The mtDNA mutation rate has been confirmed to be related to the age of onset of MELAS, and the later the onset, the lower the mutation rate of mtDNA (21). The low mutation rate of m.3243A>G in this patient may explain the patient's late onset and absence of obvious lesions in muscle biopsy.

## Conclusion

We reported a rare case of elderly onset male MELAS. Stroke-like episodes in elderly patients with MELAS are easily misdiagnosed as ischemic stroke and should be considered in the differential diagnoses when imaging findings are inconsistent with ischemic infarction. In this case, superficial temporal artery dilatation with MRA during a stroke-like migraine-like attack enriched our understanding of the clinical and imaging features of MELAS. Multimodal MRI during stroke-like episodes in MELAS can provide significant clues to a final diagnosis.

## Data availability statement

The datasets presented in this article are not readily available because of ethical and privacy restrictions. Requests to access the datasets should be directed to the corresponding author/s.

## Ethics statement

The studies involving human participants were reviewed and approved by the Ethics Committee of the First Affiliated Hospital, Guangdong Pharmaceutical University. This enrolled patient provided written, informed consent to be included in the study. All methods were performed in accordance with the relevant guidelines and regulations. The patients/participants provided their written informed consent to participate in this study.

## Author contributions

M-FH and Z-XP contributed to the conception of the study. Z-HZ, A-QL, and S-FC contributed significantly to the analysis and manuscript preparation. S-PD performed the data analyses and wrote the manuscript. All authors contributed to the article and approved the submitted version.

## Conflict of interest

The authors declare that the research was conducted in the absence of any commercial or financial relationships that could be construed as a potential conflict of interest.

## Publisher's note

All claims expressed in this article are solely those of the authors and do not necessarily represent those of their affiliated organizations, or those of the publisher, the editors and the reviewers. Any product that may be evaluated in this article, or claim that may be made by its manufacturer, is not guaranteed or endorsed by the publisher.
